# Validating a membership disclosure metric for synthetic health data

**DOI:** 10.1093/jamiaopen/ooac083

**Published:** 2022-10-11

**Authors:** Khaled El Emam, Lucy Mosquera, Xi Fang

**Affiliations:** Data Science, Replica Analytics Ltd., Ottawa, Ontario, Canada; School of Epidemiology and Public Health, University of Ottawa, Ottawa, Ontario, Canada; Research Institute, Children’s Hospital of Eastern Ontario, Ottawa, Ontario, Canada; Data Science, Replica Analytics Ltd., Ottawa, Ontario, Canada; Research Institute, Children’s Hospital of Eastern Ontario, Ottawa, Ontario, Canada; Data Science, Replica Analytics Ltd., Ottawa, Ontario, Canada

**Keywords:** synthetic data generation, data privacy, membership disclosure

## Abstract

**Background:**

One of the increasingly accepted methods to evaluate the privacy of synthetic data is by measuring the risk of membership disclosure. This is a measure of the F1 accuracy that an adversary would correctly ascertain that a target individual from the same population as the real data is in the dataset used to train the generative model, and is commonly estimated using a data partitioning methodology with a 0.5 partitioning parameter.

**Objective:**

Validate the membership disclosure F1 score, evaluate and improve the parametrization of the partitioning method, and provide a benchmark for its interpretation.

**Materials and methods:**

We performed a simulated membership disclosure attack on 4 population datasets: an Ontario COVID-19 dataset, a state hospital discharge dataset, a national health survey, and an international COVID-19 behavioral survey. Two generative methods were evaluated: sequential synthesis and a generative adversarial network. A theoretical analysis and a simulation were used to determine the correct partitioning parameter that would give the same F1 score as a ground truth simulated membership disclosure attack.

**Results:**

The default 0.5 parameter can give quite inaccurate membership disclosure values. The proportion of records from the training dataset in the attack dataset must be equal to the sampling fraction of the real dataset from the population. The approach is demonstrated on 7 clinical trial datasets.

**Conclusions:**

Our proposed parameterization, as well as interpretation and generative model training guidance provide a theoretically and empirically grounded basis for evaluating and managing membership disclosure risk for synthetic data.

## INTRODUCTION

There has been growing interest in using synthetic data generation (SDG) techniques to enable broader privacy-preserving sharing of data for secondary purposes,^1^^,^^2^ and specifically for health data.^3–13^ While patient (re-)consent is one legal basis for making data available for secondary purposes, it is often impractical to get retroactive consent under many circumstances and there is significant evidence of consent bias.^14^ Anonymization is another approach for addressing privacy concerns when making health data available for secondary analysis. However, there have been repeated claims of successful reidentification attacks on anonymized data,^15–21^ eroding public and regulator trust in this approach.^21–30^

There are multiple synthetic health datasets that are currently available to a broad research community such as: the NIH National COVID Cohort Collaborative (N3C),^31^ the CMS Data Entrepreneur’s Synthetic Public Use files,^32^ synthetic cardiovascular and COVID-19 datasets available from the CPRD in the United Kingdom,^33^^,^^34^ A&E data from NHS England,^35^ cancer data from Public Health England,^36^ a synthetic registry from the Dutch cancer registry,^37^ synthetic variants of the French public health system claims and hospital dataset (SNDS),^38^ and South Korean data from the Health Insurance Review and Assessment service (the national health insurer).^39^

The general assumption has been that synthetic data has low identity disclosure risks because there is no unique or one-to-one mapping between the records in the synthetic data with the records in the original (real) data.^40–47^ However, there are additional risks beyond identity disclosure that need to be managed for synthetic datasets: (1) attribution risk (attribute disclosure conditional on identity disclosure),^48^ and (2) membership disclosure.^49^^,^^50^ Our primary focus in this article is on evaluating membership disclosure for synthetic data.

There has been a growing literature on assessing membership disclosure risks for synthetic data.^8^^,^^49–57^ Membership disclosure is when an adversary, using the information in synthetic data, determines that a target individual was included in the real dataset used as input for SDG. Knowing that an individual was in the real data can reveal sensitive attributes about that individual if the dataset pertains to a particular disease, condition, or process. The target individual is assumed to be from the same population as the real dataset.

For example, if the real dataset pertains to a clinical study of HIV patients, membership disclosure would reveal that the target individual has HIV, or that they had participated in the study. Both would be deemed inappropriate disclosures of private information.

A broader type of membership risk, referred to as a membership inference attack, has been used to evaluate privacy risks for discriminative machine learning models.^58^^,^^59^ There are multiple reasons why membership inference attacks on machine learning models may be performed. For example, if an organization wishes to see if any of their own data was inappropriately used to train a machine learning model to detect copyright infringement or a breach of contract, or a regulator attempting to detect if some information was used without individual consent. In the context of the current study, we are only focused on membership inference attacks for the sole purpose of privacy violations. This distinction is important because the privacy purpose imposes some pragmatic constraints on these attacks.

While we are not aware of real-world membership disclosure attacks on synthetic datasets, the extensive and growing literature on the topic has highlighted the risk. From a legal and compliance perspective, it will arguably not be acceptable to share synthetic data without demonstrating low membership disclosure risks.

One proposed method for estimating membership disclosure requires the training of a shadow model,^51^ and using a discriminator, such as a random forest model, to distinguish between records in and not in the training dataset. However, this approach makes a strong assumption about the availability to the adversary of a large reference dataset from the same population as the training data,^60^ which may be difficult to meet in practice. Another strong assumption that is made is that the adversary would know the generative model details including all the parametrizations. For example, a data custodian would not generally share all of the trained weights and hyperparameters of their generative models with the data users.

Therefore, in this article, we evaluate another and more commonly used partitioning method for estimating membership disclosure risk of synthetic data, demonstrate through a theoretical and empirical analysis that its default parametrization in the literature could give inaccurate estimates of membership disclosure risk, and define a parameterization that gives the same results as the ground truth. We then provide a general benchmark to evaluate whether membership disclosure is acceptably low or not, and apply the membership disclosure metric to assess the risks for 7 clinical trial datasets.

## MATERIALS AND METHODS

### Notation

We will use the notation in Table 1.

**Table 1.** The notation used in the article

Datasets	
R	The real dataset
P	The population from which the real dataset is sampled
S	Synthetic datasets
D	The attack dataset
y	A record in the attack dataset (ie, y∈D)
$y^{\prime }$	A record in the synthetic dataset (ie, $y^{\prime }\in S$)
r	A record in the real dataset (ie, r∈R)
Dataset sizes	
n	The number of records in the real dataset (ie, n=|R|)
m	The number of records in the attack dataset (ie, m=|D|)
N	The size of the population that R is sampled from (ie, N=|P|)
t	The proportion of the attack dataset records that are in the real dataset
Hamming distance	
L	Hamming distance function
h	Hamming distance threshold

### The partitioning membership disclosure attack method

An assessment of membership disclosure is performed by the data custodian before a dataset is released. The data custodian does not have access to the population that the real dataset was sampled from, and therefore they will use an estimation procedure to compute this disclosure risk. The estimation procedure should accurately reflect the level of success that an adversary performing an attack would achieve on average. If the estimation procedure does not meet that objective, then it would not be useful for decision-making by the data custodian.

**Figure 1.**
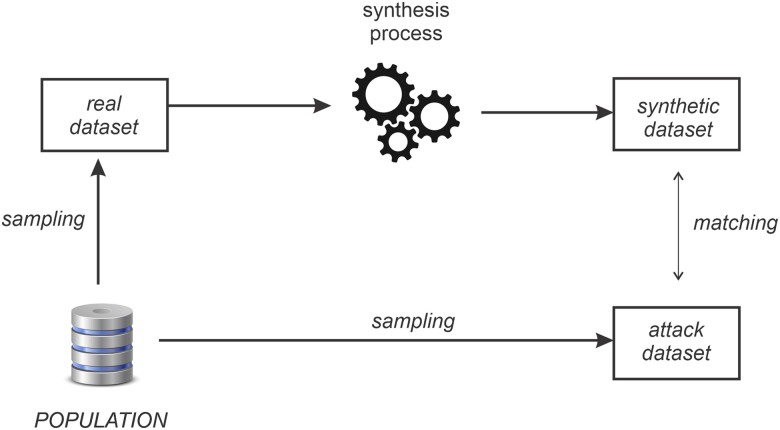
The (ground truth) process for a membership disclosure attack which accounts for the fact that the attack dataset will be sampled independently from the same population as the real dataset. The attack dataset is matched with the synthetic dataset to infer which records are in the real dataset.

Under the partitioning method a membership disclosure attack occurs when an adversary has an attack dataset that is a sample from the same population as the real dataset. The attack dataset consists of one or more target individuals that the adversary intends to compromise. Then the adversary matches records in the attack dataset with the synthetic dataset. Membership disclosure occurs when a matching record is also in the training dataset. This process is illustrated in Figure 1. A key assumption in this process is that the attack dataset is from the same population as the real dataset, otherwise there is no reason for the adversary to expect that attack dataset records would be in the real dataset.

Because the data custodian does not have access to the attack dataset nor the population, they would need to estimate this risk using a different procedure as described below. The starting assumption for the partitioning method is that the synthetic data distribution approximates the real dataset distribution.^61^ Therefore, the probability that the attack dataset belongs to the training dataset is proportional to the probability that the attack dataset belongs to the synthetic dataset. The partitioning method does not require a large reference dataset, which explains why it is the most commonly implemented in practice.^8^^,^^49^^,^^50^^,^^53^^,^^55^^,^^56^

**Figure 2.**
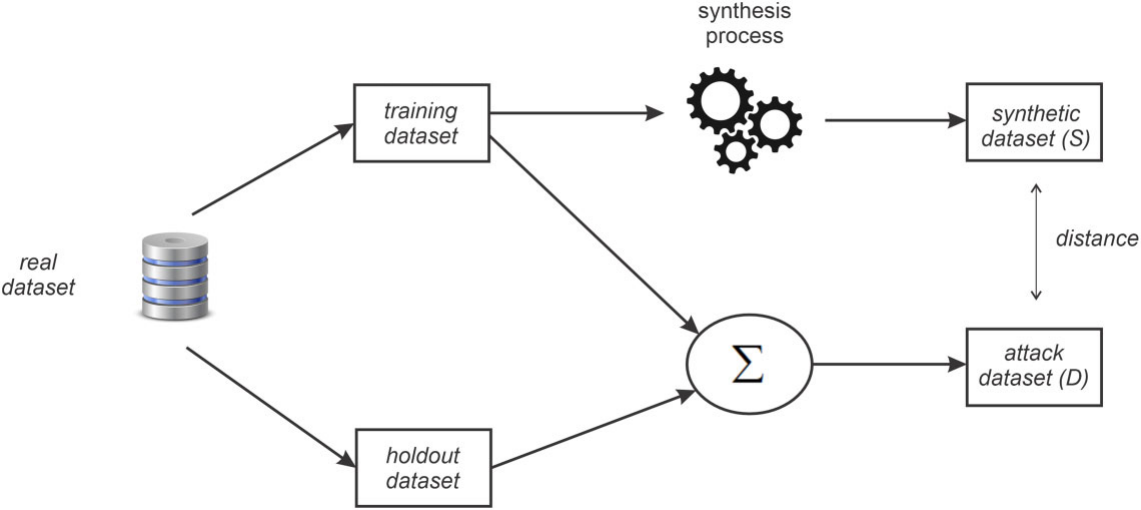
An overview of the membership disclosure evaluation process that is commonly used in the literature.

The partitioning method is illustrated in Figure 2. Here the real dataset is randomly split into 2 subsets, the training sample, and a holdout sample. The training sample is then synthesized, and a synthetic dataset is created. The holdout data provides records not used in training for inclusion in the attack dataset.

We assume that an adversary has complete information on m patients,^49^^,^^50^^,^^53^ where m×t are drawn from the training sample and m×(1−t) are drawn from the holdout sample. For example, if t=0.5 then the attack dataset is half training and half holdout. We set m=s×n were s is a sampling fraction from the real dataset. Previous work did not demonstrate a pronounced change when the sampling fraction was altered.^49^^,^^50^ Therefore, we will not consider s to be a key parameter.

We can then compute the minimum distance between every record in D and all the records in synthetic dataset S. In the literature, the distance L is measured using the Hamming distance, and a match for attack record y is considered to have occurred if $\underset{y^{\prime }}{\min}L\left(y,y^{\prime }\right)\leq h$, where h is a predefined threshold and $y^{\prime }$ is a record in the synthetic dataset. Precision and recall metrics are then computed based on the number of matched records that are in the training dataset. These can be combined through their harmonic mean into an F1 score:

(1) $$F1=\frac{2\times \text{precision}\times \text{recall}}{\text{precision}+\text{recall}}$$

The advantage of the F1 score is that it provides a single metric that can be used for decision-making and optimization during the training of the generative model.

The F1 score computed using this method is an estimate of the expected success that an adversary would have when performing the membership disclosure attack in Figure 1.

### Parameterizing the partitioning method

Previous work using the partitioning method had set t=0.5.^8^^,^^49^^,^^50^^,^^53^^,^^55^^,^^56^ In the analysis below, we show theoretically and empirically that the accuracy of the estimate of the membership disclosure F1 using the partitioning method is dependent on the value of t, and that there is a valid value of t that is consistent with the sample that an adversary would obtain when constructing an attack dataset, irrespective of the size of the attack dataset.

The real dataset is a set of records R of size n=|R|, and that a synthetic version of this dataset is generated, denoted by the set of records S. The attacker has another dataset represented by D which is the attack dataset, and we let m=|D|. Both R and D are independent random samples from the same population, which consists of the set of records P and N=|P| which is the size of the population.

With the above setup, the probability that there are k individuals in the overlap of R and D, such that k=|R∩D|, can be expressed as a hypergeometric distribution:

(2) $$pr\left(k=x\right)=\frac{\left(\begin{array}{c} N-m\\ n-x \end{array}\right)\left(\begin{array}{c} m\\ x \end{array}\right)}{\left(\begin{array}{c} N\\ n \end{array}\right)}$$

and this hypergeometric distribution has an expected value mnN. The proportion of individuals from the attack dataset that can plausibly exist in the real dataset is therefore *n/N*, which is the sampling fraction of the real dataset from the population.

This means that an adversary sampling an attack dataset from the same population as the real dataset will have an expected proportion of *t = n/N* of records in the attack dataset that are also in the real dataset. For the data custodian to correctly assess membership disclosure, that same proportion that the adversary will have should be used to give a correct estimate of the F1 score. Unless the real dataset represents 50% of the population, setting t=0.5 will not provide an attack dataset that is reflective of the expected attack dataset that an adversary would have in practice. In the empirical assessment below, we demonstrate the differences in the calculation of the F1 score from the ground truth when t=0.5.

### Empirical demonstration

In this empirical demonstration, we simulate an actual membership disclosure adversary attack on synthetic datasets as illustrated in Figure 1 and compute the F1 score for the adversary. This is the ground truth in that it provides the correct F1 success rate of a membership disclosure attack.

This ground truth simulation assumes that the adversary samples 1000 records randomly from the population and matches these records with the synthetic records. Records that match are claimed to also be in the training dataset. The claims are evaluated by computing the F1 score. This process models the adversary behavior of the membership disclosure attack as defined in the literature.

We then simulate the partitioning method illustrated in Figure 2 while varying the value of t and also compute the F1 score each time. For this simulation, we randomly select a value of t between 0 and 1 for each iteration of the simulation.

The 2 approaches are then compared to determine when they give the same results (ie, at what value of t are the F1 score values the same). This is the value of t that should be used when computing membership disclosure using the partitioning method since that is the value which gives the same result as the ground truth.

For these simulations, we ran 50 iterations for each study point where we varied the parameters as follows: (1) the t parameter was varied randomly from 0 to 1, (2) the size of the attack dataset was fixed at 1000 observations, although when we varied that parameter it had no impact on the results as we just need sufficient observations to get a stable value for F1, (3) the training dataset size was set to 5k, 15k, and 25k, (4) the Hamming distance threshold was set to 5 which is within the range of values commonly used in the literature,^61^ (5) 2 generative models were used, and (6) 4 different datasets.

The first type of generative models was a sequential tree-based synthesizer.^62^ This has been used to synthesize health and social sciences data,^63–71^ and applied in research studies on synthetic data.^63^^,^^72^^,^^73^ The second is CTGAN,^74^ which is a generative adversarial network (GAN) architecture. GANs have been applied often for the synthesis of health data.^41^^,^^49^^,^^50^^,^^53^^,^^55^^,^^75^ These 2 types of generative models are representative of those used in practice.

We used 4 datasets as the population in our simulations summarized in Table 2. These datasets were selected as they reflect heterogeneous data collection contexts including care settings, public health, and surveys. They also vary in data complexity. We set up the dataset sizes so that there is realistic variation in the sampling fractions of the real datasets that were used.

**Table 2.** The fields in the datasets used in our study (a) Ontario COVID-19 Case dataset, (b) Washington state hospital discharge database, (c) The Canadian Community Health Survey data, and (d) the Nexoid COVID-19 behavioral survey

Variable	Definitions	Variable	Definitions
(a)		(b)	
Date reported	Number of days since 1 January 2020; this variable was discretized into 20 groups	AGE	Patient age in years
Health region	34 unique regions	AMONTH	Admission month
Age group	Decades from 20 to 80+ (ordinal)	AWEEKEND	Weekend admission (Y/N)
Gender	Binary gender	DIED	Whether the patient died
Exposure	close contact, outbreak, travel, not reported	FEMALE	Sex
Case status	recovered, deceased, active	LOS	Length of stay
		ZIP	Patient ZIP code
		AYEAR	Admission year (2006 or 2007)
		DX1-DX9	Diagnosis codes
**(c)**		**(d)**	
LBSG31	Employment status over the last 12 months (full/part time)	survey_date	Date survey was administered
SMKDSTY	Type of smoker	country	Country of residence of the respondent
GEOGPRV	Province of residence	sex	Sex
DHHGAGE	Age (category)	age	Age in years
DHH_SEX	Sex	height, weight, bmi	Height (cm), weigh (kg), and BMI
DHHGMS	Marital status	blood_type	Blood type or “unknown”
DHHGLVG	Living arrangements	smoking	Amount of cigarettes smoked
DHHGHSZ	Household size	Drugs (x6)	Drugs that the respondent may be taking
GEN_08	Worked in a job or business over last 12 months	risk_infection	Calculated risk of infection with COVID-19
LBSGSOC	Occupation group	Risk_mortality	Calculated mortality risk from COVID-19
EDUDH04	Highest level of education		
SDC_8	Current student		
SDCFIMM	Immigrant or not		
SDCGCGT	Cultural or racial origin		
INCGHH	Household income		

### Interpreting the partitioning method F1 score

The F1 score is known to depend on the distribution of positive classes, which in our case is the proportion of real records in the attack dataset. This means that the F1 value by itself will not have a consistent interpretation across different datasets with varying distributions.

We propose to interpret the obtained F1 score relative to the maximum that the adversary would obtain with no background knowledge about the real dataset. The highest F1 score that can be obtained by the adversary with no background knowledge would be if they classify all of the records in the attack dataset as being in the real dataset. This value would be obtained irrespective of any synthesis—its only assumption is that the adversary has drawn a sample of targets from the same distribution as the training dataset and does not depend on the availability of a synthetic dataset. In such a case the maximum F1 score from classifying all records in the attack dataset as being in the real dataset would be:

(3) Fmax=2×nN1+nN

As N grows for a fixed n, $F_{\max}\to 0$, and $F_{\max}=1$ when n=N.

This maximum F1 score is a function of the proportion of the population that is in the real dataset. The larger that proportion the greater the success of the adversary by using this naïve strategy. That is not surprising in that the more individuals in the real dataset, claiming that a randomly selected person from the population is in the training dataset is more likely to be correct.

Note that if the adversary randomly assigns attack records to a training dataset based on a probability of 0.5, their F1 score would be lower than Equation (3). Therefore, there is no reason for an adversary to follow that suboptimal approach.

The naïve maximum value in Equation (3) will be the case even if another privacy enhancing technology was used instead of synthetic data generation. For example, if risk-based deidentification methods were used^76^ the maximum F1 score from a naïve membership disclosure attack would be the same. Under this naïve attack recall is by definition equal to 1 and precision is equal to *n/N*.

The F1 score produced using the partitioning method can be interpreted with respect to this maximum value. We define a corrected F1 score to reflect membership disclosure that is similar in construction to other metrics such as Cohen’s Kappa,^77^ and denote it with M:

(4) $$M=\frac{F-F_{\text{max}}}{1-F_{\text{max}}}$$

Note that M is undefined when $F_{\max}=1$ since no additional improvements are possible.

Previous researchers have used a 20% improvement over a naive baseline as an acceptable threshold for membership disclosure risk for synthetic data,^8^ and therefore, we can use that as a cutoff. In such a case, we would define acceptable membership disclosure risk as M≤0.2. If the M value is negative then the adversary actively matching the attack dataset with the synthetic dataset would produce results worse than the naïve approach which means that using the synthetic dataset in a membership disclosure attack reduces the relative success of the adversary.

## RESULTS

**Figure 3.**
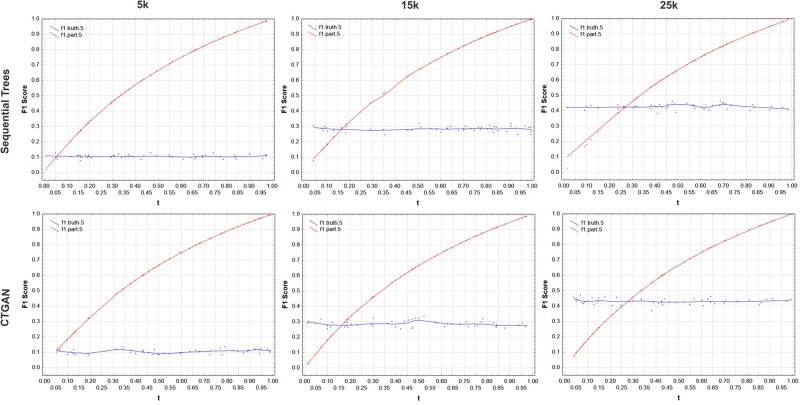
F1 score results for the COVID-19 dataset showing the ground truth from the simulation and the results using the partition method with *h* = 5. The *n/N* values for 5k, 15k, and 25k are: 0.055, 0.165, and 0.276.

**Figure 4.**
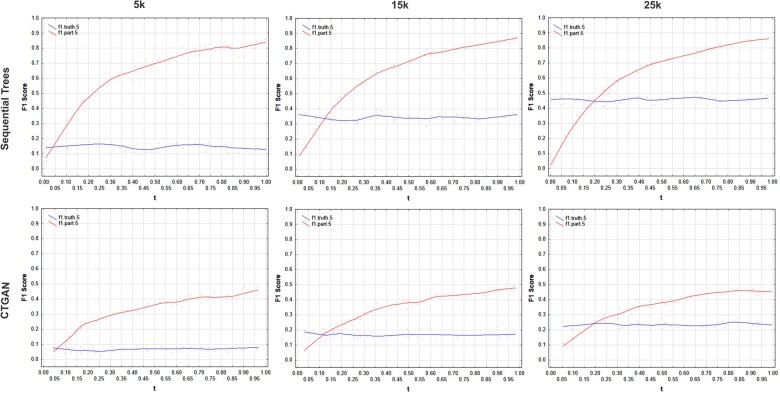
F1 score results for the Washington dataset showing the ground truth from the simulation and the results using the partition method with *h* = 5. The *n/N* values for 5k, 15k, and 25k are: 0.039, 0.116, and 0.194.

**Figure 5.**
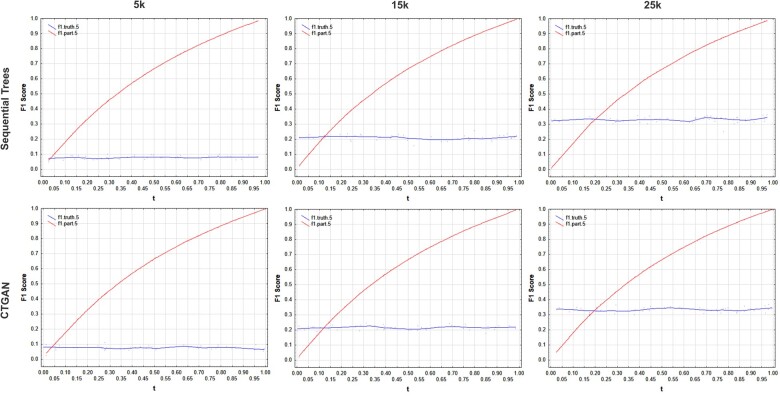
F1 score results for the CCHS dataset showing the ground truth from the simulation and the results using the partition method with *h* = 5. The *n/N* values for 5k, 15k, and 25k are: 0.039, 0.117, and 0.196.

**Figure 6.**
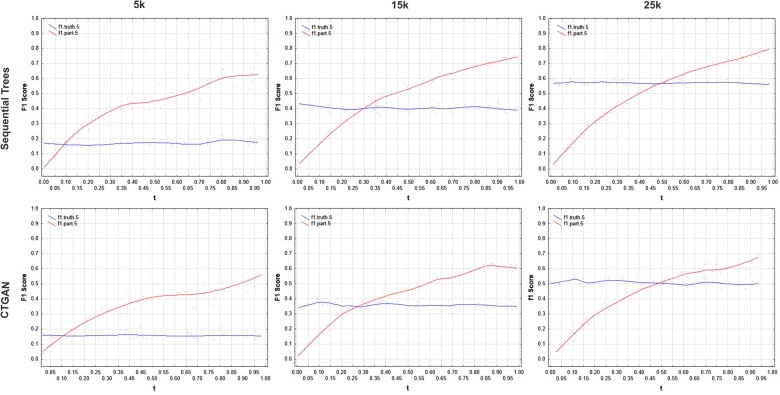
F1 score results for the Nexoid dataset showing the ground truth from the simulation and the results using the partition method with *h* = 5. The *n/N* values for 5k, 15k, and 25k are: 0.1, 0.3, and 0.5.

The graphs in Figures 3–6 show the results for the COVID, Washington, CCHS, and Nexoid datasets, respectively. The plots show the F1 score using the partitioning method as the t value is varied. The ground truth F1 score based on a simulation of a membership disclosure attack by an adversary is relatively fixed across iterations since it is not affected by the value of t.

Our results show that:

(a) The t value for the partitioning method has a nontrivial impact on the F1 score. We can see the values varying significantly across the range.
(b) The partitioning method only gives the same F1 score as the ground truth when *t = n/N* which is where the 2 lines in the plots intersect. The values of *t = n/N* are the same values where the ground truth and the partitioning method intersect in the graphs.
(c) Setting t=0.5 would not give us correct estimates of the actual membership disclosure F1 score, and sometimes the error can be quite large. Depending on whether the real data sampling fraction is above or below *t = n/N* point, the F1 score of the partitioning method can be substantially higher than or lower than the ground truth value.

These results are consistent across the datasets, generative models, and dataset sizes.

An adversary with a random sample of target individuals from the population will not achieve t=0.5 all the time and therefore always using that value will not give a true reflection of the performance of an adversary attack. When the sampling fraction is equal to 50%, the default partitioning method with t=0.5 gives the same result as the ground truth.

To further demonstrate that the correct parameterization of the partitioning method is *t = n/N*, in Table 3(a) are the mean values of the F1 score from the ground truth simulation and another simulation where we set *t = n/N* with the same number of iterations. As can be seen, the F1 score is very similar between the 2, further supporting the conclusion that this is an accurate reflection of the performance of a membership disclosure attack.

**Table 3.** F1 score results: (a) the ground truth F1 values (from the simulation) versus the F1 values estimated using the partitioning method with *t = n/N*, and (b) the mean M results on 50 iterations for the 4 datasets

**(a)**												
Dataset	Sequential trees						CTGAN					
	5k		15k		25k		5k		15k		25k	
	Act.	Est.	Act.	Est.	Act.	Est.	Act.	Est.	Act.	Est.	Act.	Est.
	F1	F1	F1	F1	F1	F1	F1	F1	F1	F1	F1	F1
COVID	0.105	0.104	0.283	0.283	0.426	0.432	0.104	0.104	0.28	0.284	0.431	0.432
Washington	0.146	0.148	0.34	0.334	0.456	0.454	0.066	0.07	0.168	0.169	0.235	0.24
CCHS	0.077	0.075	0.21	0.2	0.329	0.327	0.076	0.075	0.214	0.211	0.33	0.327
Nexoid	0.169	0.174	0.402	0.4	0.568	0.564	0.156	0.159	0.358	0.36	0.507	0.502

**(b)**						
Dataset	Sequential trees			CTGAN		
	5k	15k	25k	5k	15k	25k
COVID	−0.0002	−0.0009	−0.001	−0.0002	−0.0006	−0.0009
Washington	0.08	0.159	0.192	−0.003	−0.049	−0.122
CCHS	−0.0002	0.0003	−0.0001	<−0.0001	0.0001	−0.0001
Nexoid	−0.009	−0.114	−0.305	−0.027	−0.184	−0.492

The M values for our 3 datasets are shown in Table 3(b). All the values are below the threshold. The specific membership disclosure value is a function of the combination of dataset complexity and the generative model that is used.

## DISCUSSIONS

### Summary

Membership disclosure is considered an important privacy risk for synthetic data, and needs to be evaluated before such datasets can be used and disclosed for secondary purposes. The partitioning method for estimating membership disclosure makes reasonable assumptions about the information that an adversary has access to and is often used in the literature.

The partitioning method splits the real dataset into a training dataset and a holdout dataset. The training dataset is used to generate the synthetic data. An attack dataset is constructed with a certain proportion of it from the training dataset, and the rest from the holdout. The default in the literature is a proportion of 0.5. Then a matching exercise between the attack and synthetic dataset is performed. Matches are predicted to be in the training dataset, and the accuracy of that prediction is evaluated using an F1 score.

We showed theoretically and empirically through simulations that the proportion of training records included in the attack dataset has a nontrivial impact on the accuracy of the F1 score, and to give valid results this proportion must be equal to the sampling fraction of the real dataset from the population. If this condition is not met, the F1 score can be quite inaccurate and does not reflect the results that an adversary would obtain in practice.

An interpretable adjustment of the F1 score computed through this approach was proposed. This enables data custodians to determine whether their membership disclosure values are acceptably small or not.

The work in this article built on existing methods for assessing membership disclosure while addressing a common assumption in its calculation that has resulted in potentially inaccurate results. Our approach provides a validated and interpretable metric that can be applied on synthetic datasets.

It is necessary to provide a value for the population size. This can be defined by the prevalence of a disease in a particular geography, for example. We demonstrate this in the applications below.

### Applications

We demonstrate the application of this membership disclosure metric on 7 oncology clinical trial dataset. Given the increasing interest in making clinical trial datasets available,^78–80^ the objective was to determine what the privacy risks would be for synthetic variants, and whether these risks would be deemed acceptably small. The 7 datasets we examine are from Project Data Sphere (see https://data.projectdatasphere.org/).^81^

The population was defined as other similar trials, which is consistent with Health Canada recommendations for defining the reference population in privacy risk assessments.^79^ For each trial, we identified other trials in the same therapeutic area over the same period and with overlapping geographies from <clinicaltrials.gov>. The sequential synthesis method was used to synthesize these trial datasets.

As can be seen from the results in Table 4, the membership disclosure risks are consistently below the threshold that we had defined earlier. These suggest that sequential synthesis can be a useful generative approach for protecting the membership disclosure risks of oncology clinical trial datasets, and enable their broader sharing within the research community. This is appealing given that previous results have shown that sequential synthesis can have good utility for oncology clinical trial data^11^ and for observational datasets.^82^

**Table 4.** Summary of the oncology trials used on the analysis with the study size and the population, as well as the membership disclosure risk

Dataset	Population size (dataset size)	*M*
Trial #1 (NCT00041197): National Cancer Institute		
Tests if postsurgery receipt of imatinib could reduce the recurrence of gastrointestinal stromal tumors (GIST). Imatinib is an FDA approved protein-tyrosine kinase inhibitor for treating certain cancers of the blood cells. This drug is hypothesized to be effective against GIST as imatinib inhibits the kinase which experiences gain of function mutations in up to 90% of GIST patients.^86^ At the time of this trial the efficacy of imatinib for GIST as well as the optimal dosage for treatment of GIST was unknown.	1310 (*n* = 773)	−1.42
Trial #2 (NCT01124786): Clovis Oncology		
Most pancreatic cancer patients have advanced inoperable disease and potentially metastases. At the time of this trial the first line therapy for patients with inoperable disease was gemcitabine monotherapy. One transporter (hENT1: human equilibrative nucleoside transporter-1) has been identified as a potential predictor of successful treatment via gemcitabine. This trial compares standard gemcitabine therapy to a novel fatty acid derivative of gemcitabine. This is hypothesized to be superior to gemcitabine in metastatic pancreatic adenocarcinoma patients with low hENT1 activity as it exhibits anticancer activity independent of nucleoside transporters like hENT1, while gemcitabine seems to require nucleoside transporters for anticancer activity.	19 255 (*n* = 367)	−0.0137
Trial #3 (NCT00688740): Sanofi		
This phase 3 trial compares adjuvant anthracycline chemotherapy (fluorouracil, doxorubicin, and cyclophosphamide) with anthracycline taxane chemotherapy (docetaxel, doxorubicin, and cyclophosphamide) in women with lymph node positive early breast cancer.	21 875 (*n* = 746)	−0.034
Trial #4 (NCT00113763): Amgen		
This was a randomized Phase 3 trial examining whether panitumumab, when combined with best supportive care, improves progression-free survival among patients with metastatic colorectal cancer, compared with those receiving best supportive care alone.^87^^,^^88^ Patients included in the study had failed other chemotherapy options available at the time of the study. Participants were enrolled between 2004 and 2005.	58 381 (*n* = 370)	−0.0137
Trial #5 (NCT00460265): Amgen		
This was also a randomized Phase 3 trial on panitumumab, but among patients with metastatic and/or recurrent squamous cell carcinoma of the head and neck. The treatment group received panitumumab in addition to other chemotherapy (Cisplatin and Fluorouracil), while the control group received Cisplatin and Fluorouracil as first line therapy.^89^ Participants were enrolled between 2007 and 2009.	5868 (*n* = 520)	−0.0947
Trial #6 (NCT00119613): Amgen		
This was a randomized and blinded Phase 3 trial aimed at evaluating whether “increasing or maintaining hemoglobin concentrations with darbepoetin alfa” improves survival among patients with previously untreated extensive-stage small cell lung cancer. The treatment group received darbepoetin alfa with platinum-containing chemotherapy, whereas the control group received placebo instead of darbepoetin alfa.	16 484 (*n* = 479)	−0.0322
Trial #7 (N0147): NCCTG		
This was a randomized trial of 2686 patients with stage 3 colon adenocarcinoma that were randomly assigned to adjuvant regimens with or without Cetuximab. After resection of colon cancer, Cetuximab was added to the modified 6th version of the FOLFOX regimen including oxaliplatin plus 5-fluorouracil and leucovorin (mFOLFOX6), fluorouracil, leucovorin, and irinotecan (FOLFIRI), or a hybrid regimen consisting of mFOLFOX6 followed up by FOLFIRI.^90^ Our focus is on the secondary retrospective analysis of N0147 (the *published secondary analysis*).^91^	27 526 (*n* = 1543)	0.052

*Note*: The population includes the specific study participants. The *n* value indicates the number of trial participants for which we had data available.

This example application demonstrates how the population size was determined for clinical trial datasets. In the general context of disclosure risk estimation models, it is common to have to provide a population size value.^83–85^ In cases where determining the population size is not obvious, one default option is to use the geographic population size for the region that is covered by the dataset. If the population size is underestimated then that results in an overestimation of membership disclosure risk, and if the population size is overestimated then that results in an underestimation of membership disclosure risk. Therefore, to err on the conservative side it is preferrable to use a lower value for the population size when there is uncertainty. In the case of the default option for determining population size, this means selecting the smallest region that covers a dataset.

### Risk mitigation

For a specific dataset, it is possible to ensure that the membership disclosure risk is acceptably small by incorporating the M metric in a risk-utility loss during hyperparameter tuning of the generative model while it is being trained. The following loss metric can be used:

(5) $${\text{loss}}_{\text{RU}}=-\max\left(\left[M>0.2\right]\times \left(0.31+\frac{1}{1+e^{\left(M-1\right)}}\right),\;\left[M\leq 0.2\right]\right)\times U$$

where U is some validated utility metric^82^ and [] are Iverson brackets. This loss proportionally penalizes the utility if the membership disclosure is above the 0.2 threshold using a sigmoid function. If the risk is at or below 0.2, then the loss is equal to the utility since in that case the risk is deemed acceptable. If the risk is slightly above the 0.2 threshold then the loss is almost equal to the utility, and starts to decrease monotonically as the risk grows. The advantage of ${\text{loss}}_{\text{RU}}$ is that privacy considerations are integrated within model development rather than being a post hoc assessment.

### Limitations

In our analysis, we considered the mean results across our simulations. The variation across the iterations was largely driven by sampling variability. These results do not account for the worse case situation, but only the average performance of our membership disclosure metric.

Our membership disclosure metric is applicable to tabular data, which is consistent with the literature thus far.^8^^,^^49^^,^^50^^,^^51^^,^^53^^,^^55^^,^^56^ Future work should evaluate and extend these membership disclosure estimators to longitudinal datasets.

There are other types of privacy risks in synthetic data beyond just membership disclosure, such as attribution risks.^43^^,^^48^ In practice all privacy risks should be considered when assessing synthetic datasets.

## FUNDING

This work was partially funded by the Canada Research Chairs program through the Canadian Institutes for Health Research, a Discovery Grant RGPIN-2016-06781 from the Natural Sciences and Engineering Research Council of Canada, through a contract with the Bill and Melinda Gates Foundation, and by Replica Analytics Ltd.

## AUTHOR CONTRIBUTIONS

KEE and LM designed the study; KEE, LM, and XF performed the analysis, and wrote the paper. All authors approved the final manuscript.

## ETHICS AND CONSENT TO PARTICIPATE

This study was approved by the CHEO Research Institute Research Ethics Board protocol CHEOREB# 21/139X. All research was performed in accordance with relevant guidelines/regulations. This study only used deidentified data. Patient consent was not required by the IRB.

## Data Availability

The COVID-19 dataset is freely available from Esri Canada. The Washington hospital discharge dataset can be requested from AHRQ. The CCHS dataset can be requested from Statistics Canada under the Data Liberation Initiative. The Nexoid dataset is available from their web site: https://www.covid19survivalcalculator.com/en/research.

## References

[1] El Emam K , Mosquera L, Hoptroff R. Practical Synthetic Data Generation: Balancing Privacy and the Broad Availability of Data. Sebastopol, CA: O’Reilly Media; 2020. https://www.oreilly.com/library/view/practical-synthetic-data/9781492072737/. Accessed October 19, 2020.

[2] El Emam K , Hoptroff R. The synthetic data paradigm for using and sharing data. Cutter Executive Update 2019; 19 (6): 1–12.

[3] Foraker RE , Yu SC, Gupta A, et al Spot the difference: comparing results of analyses from real patient data and synthetic derivatives. JAMIA Open 2020; 3 (4): 557–66. 33623891 10.1093/jamiaopen/ooaa060 PMC7886551

[4] Tucker A , Wang Z, Rotalinti Y, Myles P. Generating high-fidelity synthetic patient data for assessing machine learning healthcare software. NPJ Digit Med 2020; 3 (1): 147. doi:10.1038/s41746-020-00353-9. PMC7653933 33299100

[5] Wang Z , Myles P, Tucker A. Generating and evaluating synthetic UK primary care data: preserving data utility patient privacy. In: 2019 IEEE 32nd International Symposium on Computer-Based Medical Systems (CBMS), Cordoba, Spain; June 2019: 126–131.

[6] Wang Z , Myles P, Tucker A. Generating and evaluating cross-sectional synthetic electronic healthcare data: preserving data utility and patient privacy. Comput Intell 2021; 37 (2): 819–51.

[7] Benaim AR , Almog R, Gorelik Y, et al Analyzing medical research results based on synthetic data and their relation to real data results: systematic comparison from five observational studies. JMIR Med Inform 2020; 8 (2): e16492. 32130148 10.2196/16492 PMC7059086

[8] Mendelevitch O , Lesh MD. Fidelity and privacy of synthetic medical data. arXiv:2101.08658 [cs], 2021. http://arxiv.org/abs/2101.08658. Accessed July 5, 2021.

[9] Muniz-Terrera G , Mendelevitch O, Barnes R, Lesh MD. Virtual cohorts and synthetic data in dementia: an illustration of their potential to advance research. Front Artif Intell 2021; 4: 613956. doi:10.3389/frai.2021.613956. PMC8165312 34079930

[10] Foraker R , Guo A, Thomas J, Zamstein N, Payne PRO, Wilcox A; N3C Collaborative. Analyses of original and computationally-derived electronic health record data: the National COVID Cohort Collaborative. J Med Internet Res 2021; 23 (10): e30697. 34559671 10.2196/30697 PMC8491642

[11] Azizi Z , Zheng M, Mosquera L, Pilote L, Emam KE; GOING-FWD Collaborators. Can synthetic data be a proxy for real clinical trial data? A validation study. BMJ Open 2021; 11 (4): e043497. 10.1136/bmjopen-2020-043497 PMC8055130 33863713

[12] El Emam K , Mosquera L, Jonker E, Sood H. Evaluating the utility of synthetic COVID-19 case data. JAMIA Open 2021; 4 (1): ooab012. 33709065 10.1093/jamiaopen/ooab012 PMC7936723

[13] Beaulieu-Jones BK , Wu ZS, Williams C, et al Privacy-preserving generative deep neural networks support clinical data sharing. Circ Cardiovasc Qual Outcomes 2019; 12 (7): e005122. 31284738 10.1161/CIRCOUTCOMES.118.005122 PMC7041894

[14] El Emam K , Jonker E, Moher E, Arbuckle L. A review of evidence on consent bias in research. Am J Bioeth 2013; 13 (4): 42–4. 10.1080/15265161.2013.767958 23514396

[15] de Montjoye Y-A , Hidalgo CA, Verleysen M, Blondel VD. Unique in the crowd: the privacy bounds of human mobility. Sci Rep 2013; 3: 1376. doi:10.1038/srep01376. PMC3607247 23524645

[16] de Montjoye Y-A , Radaelli L, Singh VK, Pentland AS. “Sandy” Pentland, unique in the shopping mall: on the reidentifiability of credit card metadata. Science 2015; 347 (6221): 536–9. 25635097 10.1126/science.1256297

[17] Sweeney L , Yoo JS, Perovich L, Boronow KE, Brown P, Brody JG. Re-identification risks in HIPAA safe harbor data: a study of data from one environmental health study. J Technol Sci 2017: 2017082801. https://techscience.org/a/2017082801/. Accessed March 23, 2020. PMC6344041 30687852

[18] Yoo JS , Thaler A, Sweeney L, Zang J. Risks to patient privacy: a re-identification of patients in Maine and Vermont statewide hospital data. J Technol Sci 2018: 2018100901. https://techscience.org/a/2018100901/. Accessed March 23, 2020.

[19] Sweeney L. Matching Known Patients to Health Records in Washington State Data. Cambridge, MA: Harvard University, Data Privacy Lab; 2013.

[20] Sweeney L , von Loewenfeldt M, Perry M. Saying it’s anonymous doesn’t make it so: re-identifications of ‘anonymized’ law school data. J Technol Sci 2018: 2018111301. https://techscience.org/a/2018111301/. Accessed March 23, 2020.

[21] Zewe A. Imperiled information: students find website data leaks pose greater risks than most people realize. Harvard John A. Paulson School of Engineering and Applied Sciences. January 17, 2020. https://www.seas.harvard.edu/news/2020/01/imperiled-information. Accessed March 23, 2020.

[22] Bode K. Researchers find ‘anonymized’ data is even less anonymous than we thought. Motherboard: Tech by Vice. February 3, 2020. https://www.vice.com/en_ca/article/dygy8k/researchers-find-anonymized-data-is-even-less-anonymous-than-we-thought. Accessed May 11, 2020.

[23] Clemons E. Online profiling and invasion of privacy: the myth of anonymization. *HuffPost*. February 20, 2013. https://www.huffpost.com/entry/internet-targeted-ads_b_2712586. Accessed May 11, 2020.

[24] Jee C. You’re very easy to track down, even when your data has been anonymized. *MIT Technology Review*. July 23, 2019. https://www.technologyreview.com/2019/07/23/134090/youre-very-easy-to-track-down-even-when-your-data-has-been-anonymized/. Accessed May 11, 2020.

[25] Kolata G. Your data were ‘anonymized’? These scientists can still identify you. *The New York Times*. July 23, 2019. https://www.nytimes.com/2019/07/23/health/data-privacy-protection.html. Accessed May 11, 2020.

[26] Lomas N. Researchers spotlight the lie of ‘anonymous’ data. *TechCrunch.* July 24, 2019. https://techcrunch.com/2019/07/24/researchers-spotlight-the-lie-of-anonymous-data/. Accessed May 11, 2020.

[27] Mitchell S. Study finds HIPAA protected data still at risks. *Harvard Gazette*. March 8, 2019. https://news.harvard.edu/gazette/story/newsplus/study-finds-hipaa-protected-data-still-at-risks/. Accessed May 11, 2020.

[28] Thompson SA , Warzel C. Twelve million phones, one dataset, zero privacy. *The New York Times*. December 19, 2019. https://www.nytimes.com/interactive/2019/12/19/opinion/location-tracking-cell-phone.html. Accessed May 11, 2020.

[29] Hern A. ‘Anonymised’ data can never be totally anonymous, says study. *The Guardian*. July 23, 2019. http://www.theguardian.com/technology/2019/jul/23/anonymised-data-never-be-anonymous-enough-study-finds. Accessed May 11, 2020.

[30] van der Wolk A. The (im)possibilities of scientific research under the GDPR. *Cybersecurity Law Report*. June 17, 2020. https://www.mofo.com/resources/insights/200617-scientific-research-gdpr.html. Accessed July 23, 2020.

[31] Haendel MA , Chute CG, Bennett TD, et al; N3C Consortium. The National COVID Cohort Collaborative (N3C): rationale, design, infrastructure, and deployment. J Am Med Inform Assoc 2021; 28 (3): 427–43. 32805036 10.1093/jamia/ocaa196 PMC7454687

[32] CMS. CMS 2008-2010 data entrepreneurs’ synthetic public use file (DE-SynPUF). https://www.cms.gov/Research-Statistics-Data-and-Systems/Downloadable-Public-Use-Files/SynPUFs/DE_Syn_PUF. Accessed July 17, 2022.

[33] Wang Z, Myles P, Tucker A. Generating and evaluating synthetic UK primary care data: preserving data utility & patient privacy. In: 2019 IEEE 32nd International Symposium on Computer-Based Medical Systems (CBMS); 2019: 126–31. https://ieeexplore-ieee-org/document/8787436. Accessed August 31, 2019.

[34] Synthetic data at CPRD. Medicines & Healthcare products Regulatory Agency, United Kingdom. https://www.cprd.com/content/synthetic-data. Accessed September 24, 2020.

[35] NHS England. A&E synthetic data. https://data.england.nhs.uk/dataset/a-e-synthetic-data. Accessed July 16, 2022.

[36] The Simulacrum. *The Simulacrum*. https://simulacrum.healthdatainsight.org.uk/. Accessed November 27, 2021.

[37] Synthetic dataset. integraal kankercentrum Nederland 2021. https://iknl.nl/en/ncr/synthetic-dataset2021. Accessed November 20, 2021.

[38] SNDS synthétiques. Systeme national des donnees de sante, 2021. https://documentation-snds.health-data-hub.fr/formation_snds/donnees_synthetiques/. Accessed January 20, 2022.

[39] #opendata4covid19 Website User Manual. Ministry of Health and Welfare; Health Insurance Review & Assessment Service (HIRA), April 2020. https://rtrod-assets.s3.ap-northeast-2.amazonaws.com/static/tools/manual/COVID-19+website+manual_v2.1.pdf. Accessed April 8, 2020.

[40] Reiter JP. New approaches to data dissemination: a glimpse into the future (?). Chance 2004; 17 (3): 11–5.

[41] Park N , Mohammadi M, Gorde K, Jajodia S, Park H, Kim Y. Data synthesis based on generative adversarial networks. Proc VLDB Endow 2018; 11 (10): 1071–83.

[42] Hu J. Bayesian estimation of attribute and identification disclosure risks in synthetic data. arXiv:1804.02784 [stat], April 2018. http://arxiv.org/abs/1804.02784. Accessed March 15, 2019.

[43] Taub J , Elliot M, Pampaka M, Smith D. Differential correct attribution probability for synthetic data: an exploration. In: Privacy in Statistical Databases. Cham: Springer; 2018: 122–37.

[44] Hu J , Reiter JP, Wang Q. Disclosure risk evaluation for fully synthetic categorical data. In: Privacy in Statistical Databases. Cham: Springer; 2014: 185–99.

[45] Wei L , Reiter JP. Releasing synthetic magnitude microdata constrained to fixed marginal totals. Stat J IAOS 2016; 32 (1): 93–108.

[46] Ruiz N , Muralidhar K, Domingo-Ferrer J. On the privacy guarantees of synthetic data: a reassessment from the maximum-knowledge attacker perspective. In: Privacy in Statistical Databases. Cham: Springer; 2018: 59–74.

[47] Reiter JP. Releasing multiply imputed, synthetic public use microdata: an illustration and empirical study. J R Stat Soc Ser A Stat Soc 2005; 168 (1): 185–205.

[48] Emam KE , Mosquera L, Bass J. Evaluating identity disclosure risk in fully synthetic health data: model development and validation. J Med Internet Res 2020; 22 (11): e23139. https://www.jmir.org/2020/11/e23139. Accessed October 13, 2020. 10.2196/23139 PMC7704280 33196453

[49] Choi E , Biswal S, Malin B, Duke J, Stewart WF, Sun J. Generating multi-label discrete patient records using generative adversarial networks. In: Machine Learning for Healthcare Conference; Maastricht, NL: MLResearchPress; 2017; vol. 68, pp. 286–305. http://proceedings.mlr.press/v68/choi17a/choi17a.pdf. Accessed July 11, 2019.

[50] Zhang Z , Yan C, Mesa DA, Sun J, Malin BA. Ensuring electronic medical record simulation through better training, modeling, and evaluation. J Am Med Inform Assoc 2020; 27 (1): 99–108. doi:10.1093/jamia/ocz161. 31592533 PMC6913223

[51] Stadler T , Oprisanu B, Troncoso C. Synthetic data—Anonymisation Groundhog Day. *arXiv:2011.07018 [cs]*, v6 published January 2022. http://arxiv.org/abs/2011.07018. Accessed February 1, 2022.

[52] Torfi A , Fox EA. CorGAN: correlation-capturing convolutional generative adversarial networks for generating synthetic healthcare records. *arXiv:2001.09346 [cs, stat]*, March 2020. http://arxiv.org/abs/2001.09346. Accessed July 24, 2020.

[53] Yan C , Zhang Z, Nyemba S, Malin BA. Generating electronic health records with multiple data types and constraints. *arXiv:2003.07904 [cs, stat]*, March 2020. http://arxiv.org/abs/2003.07904. Accessed June 28, 2020. PMC8075510 33936510

[54] Zhang Z , Yan C, Lasko TA, Sun J, Malin BA. SynTEG: a framework for temporal structured electronic health data simulation. J Am Med Inform Assoc 2020; 28 (3): 596–604. 10.1093/jamia/ocaa262 PMC7936402 33277896

[55] Goncalves A , Ray P, Soper B, Stevens J, Coyle L, Sales AP. Generation and evaluation of synthetic patient data. BMC Med Res Methodol 2020; 20 (1): 108. 32381039 10.1186/s12874-020-00977-1 PMC7204018

[56] Chen D , Yu N, Zhang Y, Frtiz M. GAN-leaks: a taxonomy of membership inference attacks against generative models. In: ACM SIGSAC Conference on Computer and Communications Security, USA Virtual; New York: Association for Computing Machinery; November 2020. https://dl.acm.org/doi/10.1145/3372297.3417238. Accessed January 9, 2022.

[57] Hilprecht B , Härterich M, Bernau D. Monte Carlo and reconstruction membership inference attacks against generative models. Proc Priv Enh Technol 2019; 2019 (4): 232–49.

[58] Shokri R , Stronati M, Song C, Shmatikov V. Membership inference attacks against machine learning models. In: 2017 IEEE Symposium on Security and Privacy (SP); California: IEEE Computer Society; May 2017: 3–18. doi:10.1109/SP.2017.41.

[59] Truex S , Liu L, Gursoy ME, Yu L, Wei W. Demystifying membership inference attacks in machine learning as a service. IEEE Trans Serv Comput 2021; 14 (6): 2073–89.

[60] van der Ploeg T , Austin PC, Steyerberg EW. Modern modelling techniques are data hungry: a simulation study for predicting dichotomous endpoints. BMC Med Res Methodol 2014; 14 (1): 137. 25532820 10.1186/1471-2288-14-137 PMC4289553

[61] Sun H , Zhu T, Zhang Z, Jin D, Xiong P, Zhou W. Adversarial attacks against deep generative models on data: a survey. IEEE Trans Knowl Data Eng 2021: 1–1.

[62] Emam KE , Mosquera L, Zheng C. Optimizing the synthesis of clinical trial data using sequential trees. J Am Med Inform Assoc 2020; 28 (1): 3. 10.1093/jamia/ocaa249 PMC7810457 33186440

[63] Drechsler J , Reiter JP. An empirical evaluation of easily implemented, nonparametric methods for generating synthetic datasets. Comput Stat Data Anal 2011; 55 (12): 3232–43.

[64] Arslan RC , Schilling KM, Gerlach TM, Penke L. Using 26,000 diary entries to show ovulatory changes in sexual desire and behavior. J Pers Soc Psychol 2021; 121 (2): 410–31. doi:10.1037/pspp0000208. 30148371

[65] Bonnéry D , Feng Y, Henneberger AK, et al The promise and limitations of synthetic data as a strategy to expand access to state-level multi-agency longitudinal data. J Res Educ Eff 2019; 12 (4): 616–47.

[66] Sabay A , Harris L, Bejugama V, Jaceldo-Siegl K. Overcoming small data limitations in heart disease prediction by using surrogate data. SMU Data Science Review 2018; 1 (3): 25. https://scholar.smu.edu/datasciencereview/vol1/iss3/12.

[67] Freiman M , Lauger A, Reiter J. Data synthesis and perturbation for the American Community Survey at the U.S. Census Bureau. US Census Bureau, Working Paper; 2017. https://www.census.gov/library/working-papers/2018/adrm/formal-privacy-synthetic-data-acs.html. Accessed February 24, 2020.

[68] Nowok B. Utility of synthetic microdata generated using tree-based methods. In: UNECE Statistical Data Confidentiality Work Session; October 5–7, 2015; Helsinki. https://unece.org/statistics/events/SDC2015. Accessed February 24, 2020.

[69] Raab GM , Nowok B, Dibben C. Practical data synthesis for large samples. J Priv Confid 2018; 7 (3): 67–97.

[70] Nowok B , Raab GM, Dibben C. Providing bespoke synthetic data for the UK longitudinal studies and other sensitive data with the synthpop package for R 1. Stat J IAOS 2017; 33 (3): 785–96.

[71] Quintana DS. A synthetic dataset primer for the biobehavioural sciences to promote reproducibility and hypothesis generation. eLife 2020; 9: e53275. doi:10.7554/eLife.53275. PMC7112950 32159513

[72] Little C , Elliot M, Allmendinger R, Samani S. Generative adversarial networks for synthetic data generation: a comparative study. Geneva: United Nations Economic Commission for Europe; 2021. https://unece.org/statistics/documents/2021/12/working-documents/generative-adversarial-networks-synthetic-data. Accessed January 17, 2022.

[73] Taub J , Elliot M, Sakshaug W. The impact of synthetic data generation on data utility with application to the 1991 UK samples of anonymised records. Trans Data Priv 2020; 13 (1): 1–23.

[74] Xu L , Skoularidou M, Cuesta-Infante A, Veeramachaneni K. Modeling tabular data using conditional GAN. Adv Neural Inf Process Syst 2019; 32: 7335–45. https://papers.nips.cc/paper/2019/hash/254ed7d2de3b23ab10936522dd547b78-Abstract.html. Accessed October 2, 2021.

[75] Chin-Cheong K , Sutter T, Vogt JE. Generation of heterogeneous synthetic electronic health records using GANs. In: Workshop on Machine Learning for Health (ML4H) at the 33rd Conference on Neural Information Processing Systems (NeurIPS 2019), Vancouver, BC; December 2019.

[76] El Emam K. Guide to the De-Identification of Personal Health Information. Auerbach: CRC Press; 2013.

[77] Fleiss JL , Levin B, Paik MC. Statistical Methods for Rates & Proportions. 3rd ed. Hoboken, NJ: Wiley-Interscience; 2003.

[78] European Medicines Agency. European Medicines Agency policy on publication of data for medicinal products for human use: Policy 0070. October 2, 2014. http://www.ema.europa.eu/docs/en_GB/document_library/Other/2014/10/WC500174796.pdf. Accessed July 11, 2019.

[79] Health Canada. Public release of clinical information in drug submissions and medical device applications. *Health Canada*, March 2017. https://www.canada.ca/en/health-canada/programs/public-release-clinical-information-drug-submissions-medical-device-applications.html. Accessed January 5, 2022.

[80] Strom BL, Buyse M, Hughes J, Knoppers BM. Data Sharing, Year 1 — Access to Data from Industry-Sponsored Clinical Trials. *N Engl J Med* 2014; 371 (22): 2052–4. http://www.nejm.org/doi/pdf/10.1056/NEJMp1411794. Accessed February 26, 2015. 10.1056/NEJMp1411794 25317745

[81] CEO Life Sciences Consortium. Share, integrate & analyze cancer research data. Project Data Sphere. Morrisville, NC. https://projectdatasphere.org/projectdatasphere/html/home. Accessed July 11, 2019.

[82] Emam KE , Mosquera L, Fang X, El-Hussuna A. Utility metrics for evaluating synthetic health data generation methods: validation study. JMIR Med Inform 2022; 10 (4): e35734. 35389366 10.2196/35734 PMC9030990

[83] Jiang Y , Mosquera L, Jiang B, Kong L, Emam KE. Measuring re-identification risk using a synthetic estimator to enable data sharing. PLoS One 2022; 17 (6): e0269097. 35714132 10.1371/journal.pone.0269097 PMC9205507

[84] Rocher L , Hendrickx JM, de Montjoye Y-A. Estimating the success of re-identifications in incomplete datasets using generative models. Nat Commun 2019; 10 (1): 1–9. 31337762 10.1038/s41467-019-10933-3 PMC6650473

[85] Dankar F , Emam KE, Neisa A, Roffey T. Estimating the re-identification risk of clinical data sets. BMC Med Inform Decis Mak 2012; 12: 66. 22776564 10.1186/1472-6947-12-66 PMC3583146

[86] Sarlomo-Rikala M , Kovatich AJ, Barusevicius A, Miettinen M. CD117: a sensitive marker for gastrointestinal stromal tumors that is more specific than CD34. Mod Pathol 1998; 11 (8): 728–34. 9720500

[87] Amado RG , Wolf M, Peeters M, et al Wild-type KRAS is required for panitumumab efficacy in patients with metastatic colorectal cancer. J Clin Oncol 2008; 26 (10): 1626–34. 18316791 10.1200/JCO.2007.14.7116

[88] Van Cutsem E , Peeters M, Siena S, et al Open-label phase III trial of panitumumab plus best supportive care compared with best supportive care alone in patients with chemotherapy-refractory metastatic colorectal cancer. J Clin Oncol 2007; 25 (13): 1658–64. 17470858 10.1200/JCO.2006.08.1620

[89] Vermorken JB , Stöhlmacher-Williams J, Davidenko I, et al Cisplatin and fluorouracil with or without panitumumab in patients with recurrent or metastatic squamous-cell carcinoma of the head and neck (SPECTRUM): an open-label phase 3 randomised trial. Lancet Oncol 2013; 14 (8): 697–710. 23746666 10.1016/S1470-2045(13)70181-5

[90] Alberts SR , Sargent DJ, Nair S, et al Effect of oxaliplatin, fluorouracil, and leucovorin with or without cetuximab on survival among patients with resected stage III colon cancer: a randomized trial. JAMA 2012; 307 (13): 1383–93. 22474202 10.1001/jama.2012.385 PMC3442260

[91] Dahdaleh FS , Sherman SK, Poli EC, et al Obstruction predicts worse long-term outcomes in stage III colon cancer: a secondary analysis of the N0147 trial. Surgery 2018; 164 (6): 1223–9. 30297240 10.1016/j.surg.2018.06.044

